# Spatial Dynamics and High Risk Transmission Pathways of Poliovirus in Nigeria 2001-2013

**DOI:** 10.1371/journal.pone.0163065

**Published:** 2016-09-26

**Authors:** Tara D. Mangal, R. Bruce Aylward, Faisal Shuaib, Michael Mwanza, Muhammed A. Pate, Emmanuel Abanida, Nicholas C. Grassly

**Affiliations:** 1 Department of Infectious Disease Epidemiology, Imperial College London, London, United Kingdom; 2 World Health Organization (WHO), Geneva, Switzerland; 3 National Primary Healthcare Development Agency (NPHCDA), Abuja, Nigeria; 4 World Health Organization (WHO), Amman, Jordan; 5 Duke Global Health Institute, Durham, North Carolina, United States of America; Fundacao Oswaldo Cruz, BRAZIL

## Abstract

The polio eradication programme in Nigeria has been successful in reducing incidence to just six confirmed cases in 2014 and zero to date in 2015, but prediction and management of future outbreaks remains a concern. A Poisson mixed effects model was used to describe poliovirus spread between January 2001 and November 2013, incorporating the strength of connectivity between districts (local government areas, LGAs) as estimated by three models of human mobility: simple distance, gravity and radiation models. Potential explanatory variables associated with the case numbers in each LGA were investigated and the model fit was tested by simulation. Spatial connectivity, the number of non-immune children under five years old, and season were associated with the incidence of poliomyelitis in an LGA (all *P* < 0.001). The best-fitting spatial model was the radiation model, outperforming the simple distance and gravity models (likelihood ratio test P < 0.05), under which the number of people estimated to move from an infected LGA to an uninfected LGA was strongly associated with the incidence of poliomyelitis in that LGA. We inferred transmission networks between LGAs based on this model and found these to be highly local, largely restricted to neighbouring LGAs (e.g. 67.7% of secondary spread from Kano was expected to occur within 10 km). The remaining secondary spread occurred along routes of high population movement. Poliovirus transmission in Nigeria is predominantly localised, occurring between spatially contiguous areas. Outbreak response should be guided by knowledge of high-probability pathways to ensure vulnerable children are protected.

## Introduction

The World Health Assembly determined in 1988 that paralytic poliomyelitis would be eliminated from the 125 infected countries by the year 2000 [1]. Despite great successes in eliminating polio in Europe, the Americas and parts of Asia by the Global Polio Eradication Initiative (GPEI), a few remaining endemic countries continue to support poliovirus transmission. Disease burden from the three wild poliovirus serotypes has fallen from 350,000 cases worldwide in 1988 to just 122 cases in 2014 and since 2012, wild poliovirus types 2 and 3 have not been detected [2]. The international spread of poliovirus from the endemic countries to polio-free countries constitutes a “public health emergency of international concern” but continued progress in interrupting transmission and protecting the most vulnerable makes global eradication a realistic prospect [3]. The three endemic countries, Nigeria, Pakistan and Afghanistan, have seen remarkable reductions in case numbers over the past five years; indeed by June 2016 Nigeria had reached almost two years without reporting a single case due to wild poliovirus.

In the last decade, outbreaks of poliomyelitis in Nigeria have been limited to the northern states, predominantly around a few, key districts which have suffered the greatest burden of disease. These districts were often the target of intensive “mop-up” campaigns with oral poliovirus vaccine (OPV) in addition to the supplementary and routine immunisation programmes conducted in all at-risk areas. Despite this focussed vaccination strategy, cases continued to appear, aided by sub-optimal population immunity. The recent successes in the global polio eradication programme in reducing case numbers in Nigeria have brought the goal of eradication closer. Serotypes 2 and 3 wild poliovirus are no longer circulating in Nigeria and serotype 1 wild poliovirus caused just six cases of paralytic poliomyelitis in 2014–2015, compared with 53 cases in 2013 [4]. Cases due to type 2 circulating vaccine-derived poliovirus still pose a threat to the eradication programme and identifying networks of high probability transmission is essential to limit the spread of these emerging viruses. Poliovirus transmission can be difficult to track, as the majority of carriers are asymptomatic and those that do present with symptoms experience a long incubation period [5].

Spatial models have long been used to describe the transmission of infectious diseases and rely on an underlying knowledge of population movements and their relationship to disease transmission [6–8]. Several studies have implemented spatial models to describe subnational transmission of polio in Nigeria. Analysis of the serotype 2 vaccine-derived poliovirus outbreak in Nigeria during 2005–2008 using partially-observed transmission networks highlighted the difficulties in applying spatial models to a disease with a low case: infection ratio such as polio [9]. Such a low sampling rate precluded estimation of the direct ancestors for cases and reconstruction of the underlying transmission network. Certain risk factors, such as measures of immunity, presence (and distance) of cases in neighbouring districts and population density can predict outbreaks at a subnational level [10–12]. Extending this type of analysis to predict pathways linking districts along which poliovirus transmission is highly likely to occur by incorporating human mobility patterns would provide a valuable tool for outbreak response. This would enable key planning and policy questions currently faced by the GPEI to be addressed, for example, if a district reports a case of poliomyelitis, is it feasible to stop the outbreak by immunising only in neighbouring districts?

Human mobility patterns can be captured using a number of sources such as census records or transport data, but in the absence of detailed data on population movement, as is the case in Nigeria, we rely on spatial coupling models linking pairs of locations to predict pathways of highly frequent travel. In the simplest case, spatial models assume that the connectivity between populations is a function of the distance between them, overlooking the fact that population movement tends to be higher between more populous areas [13]. Density-dependent gravity models have previously been used to describe transmission pathways of highly contagious airborne viruses such as measles and influenza which have high case: infection ratios [14–16]. However, it is unknown whether a gravity model can accurately describe the spatial dynamics of poliovirus, an enterovirus spread via the faecal-oral route [5]. The radiation model, first developed to describe radiation and absorption processes in particle physics, captures “fluxes” between population centres by considering daily commuting patterns driven by job-seeking behaviour. This model has been shown to outperform the gravity model in capturing patterns of movement on various timescales, from hourly trips extracted from mobile phone records to long-term migration patterns [17].

Here we use data on poliomyelitis incidence in Nigeria during 2001–2013 to examine the spread of poliovirus and identify potential risk factors such as population immunity, seasonality and population density. We also explore potential pathways with a high probability of poliovirus transmission and discuss the implications of our findings for the appropriate scale of the vaccination response.

## Methods

### Data

Nigeria is divided into 774 local government areas (LGAs), each with a mean area of 1,165km^2^ (SD 1,414 km^2^) and mean population size in 2006 of 177,363 (SD 86,734) [18]. We used the Euclidean distance between the centroids of each LGA pair (*d*_*ij*_) and estimated the area of each LGA using the Spatial Statistics toolbox in ArcMap 10.

The incidence data used in this analysis was collected during routine acute flaccid paralysis (AFP) surveillance in Nigeria between January 2001 and November 2013. All children with AFP who are reported to a health facility undergo clinical and laboratory investigations, including a verbal report of vaccination history (typically from the caregiver) [19]. We defined a poliomyelitis case as any child with clinically confirmed AFP and at least one stool sample positive for wild poliovirus. We considered serotype 1 cases only, as no cases of poliomyelitis due to serotype 3 have been detected in Nigeria since 16^th^ November 2012. Four OPV formulations are currently licensed for use in Nigeria, trivalent OPV, two monovalent OPVs and a bivalent OPV. We therefore estimated the numbers of each type of OPV received by a child with AFP by multiplying the total number of doses reported during the initial case investigation by the proportion of supplementary immunisation activities (SIA) in their LGA between the child’s date of birth and onset of paralysis that used the corresponding type of OPV (see Mangal *et al*, Supplementary Appendix for details) [12]. We assumed that all doses were received via SIAs rather than through routine immunisation, as routine coverage in key areas is low and highly heterogeneous [20].

We calculated the size of the susceptible population aged under 15 years using census data and sliding window estimates of vaccine-induced population immunity. The majority of reported non-polio AFP cases are aged under five years and we use this age group to estimate immunity. The individual probability of protection against poliomyelitis for non-polio AFP cases under five years of age was estimated using the efficacy of the trivalent, bivalent and monovalent vaccines (*v*) which differs for northern and southern LGAs [12] and the number of doses received (*x*):

$$1-{\left(1-v_{t}\right)}^{x_{t}}\;{\left(1-v_{b}\right)}^{x_{b}}\;{\left(1-v_{m}\right)}^{x_{m}}$$ (1)

This was used to calculate an average probability of protection for each LGA. Uncertainty in vaccine efficacy estimates was incorporated by randomly sampling 1000 times from a multivariate normal distribution of efficacy estimates and re-estimating individual and population immunity. We then used the median population immunity value multiplied by the population size under 15 years of age to derive the susceptible population size.

### Statistical analysis

We fitted a generalised linear mixed-effects model with an over-dispersed Poisson error structure to the number of cases over a six month interval in a given LGA. We included a random effects term in the intercept for observations from the same LGA, to allow for the correlated nature of these observations [21]. Population density derived from census data [18] (S1 Fig), seasonality, population size and susceptible population size were included as predictor variables in an univariate analysis, then significant terms (P < 0.05 in the Poisson mixed-effects model) were retained for a multivariate regression. The spatial model components (distance, gravity and radiation) were examined first as separate predictor variables in the univariate analysis, then as part of a force of infection term described below. The force of infection is a composite parameter, including exposure to infection from within the district (captured by past incidence of poliomyelitis), exposure to infection resulting from cases in neighbouring districts (incorporating spatial connectivity) and a background level of infection reflecting long distance travel and importation of infection nationwide. Consequently, the model formulation for the expected number of cases (*μ*_*i*_) in LGA *i* with a force of infection (*λ*_*i*_), potential explanatory variables such as population density and seasonality (*X*_*i1*_ … *X*_*iq*_) and a random effects term (*ε*_*i*_) was:

$$\log\left(\mu_{i,t}\right)\sim \alpha +\beta_{1}\lambda_{i,t}+\beta_{2}X_{i1}+\ldots +\beta_{q}X_{iq}+\varepsilon_{i}$$ (2)

The transmission intensity (*λ*_*i*_) estimated the force of infection acting on LGA (*i*) within each six month time-period. The transmission variable (*λ*_*i*_) at time *t+1* was described by:

$$\begin{array}{l} \lambda_{i,t+1}=\alpha_{1}I_{i,t}+\alpha_{2}\sum_{j,j\neq i}I_{j,t}S_{ij}+\alpha_{3}\sum_{j,j\neq i}I_{j,t}\\ S_{ij}\in D_{ij},G_{ij},T_{ij} \end{array}$$ (3)

The incidence (*I*) refers to the number of virologically confirmed poliomyelitis cases in the preceding time period *t*. The first term of the transmission variable (*I*_*i*,*t*_) refers to new infections as a result of ongoing transmission within a LGA. The second includes the spatial component (*I*_*j*,*t*_ *S*_*ij*_) describing the connectivity between LGA *i* and *j*, which may be one of three spatial models detailed below, and the third term (*I*_*j*,*t*_) denotes a background force of infection experienced equally by all LGAs, independent of distance or population size. This background risk of infection may partly explain the appearance of cases in previously unaffected areas, far from LGAs currently experiencing symptomatic cases. The parameters *α*_*1*_, *α*_*2*_, and *α*_*3*_ in Eq 3 were estimated simultaneously with the beta coefficients in Eq 2. An optimisation algorithm was used to find the optimum parameter values which maximised the likelihood returned by the over-dispersed Poisson model.

### Spatial component

To explore the effect of population movement on the risk of infection, we compared three spatial models to describe the connectivity between LGAs. Each of *N* LGA administrative regions (*i*) forms one unit (*N* = 774) with a constant population size *n*_*i*_ and distance (*d*_*ij*_) between all LGA pairs. The strength of connectivity between LGAs *i* and *j* was described in three different ways. Firstly, we used a density-independent distance model (*D*_*ij*_), with spatial coupling between two locations depending only on the distance between them, independent of the population sizes (Eq 3) [22]. The strength of connectivity was described by a power law *γ*, optimised during the multiple regression analysis.

$$D_{ij}=\frac{1}{d_{ij}^{\gamma }}$$ (4)

Secondly, we used a gravity model (*G*_*ij*_), which allows for a flexible dependence between each city pair *i* and *j*, driven by their respective population sizes (*n*_*i*_ and *n*_*j*_) and the distance (*d*_*ij*_) between them (Eq 4). The parameters *γ*, *μ* and *ν* are optimised exponents for the distance, source populations and destination populations respectively and the population sizes are in units of 100,000 people. The distance exponent will be low if disease spread is highly geographically concentrated.

$$G_{ij}=\frac{n_{i}^{\mu }n_{j}^{\nu }}{d_{ij}^{\gamma }}$$ (5)

Thirdly, we used a radiation model (*T*_*ij*_), predicting mobility patterns using a diffusion model (Eq 5) [17]. The radiation model predicts that the population movement between LGAs *i* and *j* is dependent on the population sizes *n*_*i*_ and *n*_*j*_ and the population within a circle of radius equal to the distance between the two populations *s*_*ij*_ centred on LGA *i* [17]. We included two elements in this analysis, one describing commuter flow from uninfected LGA *i* to infected LGA *j*, and the second describing commuter flow from infected LGA *j* to *i*, since these terms are not symmetric.

$$T_{ij}=T_{i}\frac{n_{i}^{}n_{j}^{}}{\left(n_{i}+s_{ij}^{}\right)\left(n_{i}+n_{j}+s_{ij}\right)}$$ (6)

By combining expected population movements with incidence in a given time period $\sum_{j,j\neq i}I_{j,t}S_{ij}$, it is possible to connect pairs of LGA based on the likelihood of infection travelling between them. We estimated these high probability transmission pathways by aggregating incidence and movement patterns over six year periods (and additionally over one-year periods, S2–S4 Figs).

### Model fitting

The unknown parameters were estimated jointly by optimisation to find the best parameter set which maximised the log likelihood returned by the mixed-effects regression model. Five sets of initial values within a credible range were chosen to explore the sensitivity of convergence to the starting values. All numerical optimisations were performed in R (version 3.0.2) using the Broyden, Fletcher, Goldfarb and Shanno variable metric algorithm with box constraints and the likelihood of the over-dispersed Poisson mixed-effects regression model calculated using the lme4 package.

The fit of the model was tested by simulation. Firstly, we derived the mean number of LGAs predicted to experience at least one case from 1000 simulated datasets using the fitted model, comparing it to observed data using R^2^. Secondly, we simulated 1000 datasets for each time period using data up to that period. The accuracy of the model in identifying the number and locations of cases during each six month period was assessed using a receiver operator characteristic (ROC) curve which plots the true positive rate against the false positive rate at various thresholds. The area under the curve (AUC) was calculated for each simulated set of responses and the average value is presented. Finally, to predict the number and distribution of cases occurring as a result of one poliomyelitis case during the high season months of April to September 2013, given the conditions in each LGA during this time period (for example, the size of the susceptible population derived from vaccination coverage estimates during this time), we fitted the model to data up to September 2013. The resulting model was used to predict case numbers in the following six months, under the assumption that population immunity in each LGA remained the same. We used Kano Municipal Area, the capital of Kano state in North Central Nigeria, as an example here as it had historically sustained uninterrupted transmission of wild poliovirus. Kano Municipal Area was classified as having a high risk of polio outbreaks in 2014; 33 of 44 LGAs within the state are high risk or very high risk LGAs [23]. From the geographic distribution of resulting cases, we estimated the size of the vaccination response needed to give each child in at-risk LGAs one booster dose of OPV following an outbreak in Kano Municipal Area. We classified districts as “at-risk” if their predicted number of cases was greater than 0.5.

Institutional ethics approval and individual informed consent were not sought because this is a retrospective study using anonymised national surveillance data collected routinely by the Government of Nigeria.

## Results

Univariate analyses of all spatial components and other potential explanatory variables showed no significant association of population density or population size under 15 years of age (*P* = 0.276 and *P* = 0.159 respectively) with the risk of outbreaks (Table 1). The force of infection term, irrespective of spatial model, the size of the susceptible population (based on vaccine-induced immunity) and high season (months April-September) versus low season (all *P* < 0.05) were associated with the incidence of poliomyelitis.

**Table 1:** Univariate Analysis of Potential Explanatory Variables Associated With Risk of Poliomyelitis Cases in Nigeria, 2001–2013 Using a Poisson Mixed Effects Regression Model.

	Coefficient(95% CI)	P-value	Log likelihood
Season(high versus low)	0.56(0.41–0.70)	<0.001	-5930.286
Population density (km^-2^)	0.07(-0.05–0.18)	0.276	-5960.887
Population size <15 years	-2.34e-6(-5.60e-6–9.19e-7)	0.159	-6003.284
Susceptible population size <15 years	2.09e-5(1.87e-5–2.31e-5)	<0.001	-5805.839
**Spatial components**			
Distance model	0.79(0.55–1.03)	<0.001	-5941.396
Gravity model	0.01(0.01–0.02)	<0.001	-5956.154
Radiation model	0.05(0.05–0.06)	<0.001	-5894.627

Coefficients obtained by maximum likelihood are presented with 95% confidence intervals.

The density-independent distance model and density-dependent gravity models offered only a marginal improvement over the non-spatial model (likelihood ratio test, both *P* < 0.05, Table 2). The radiation model clearly provides a better fit to the data (log likelihood -5023 compared with -5219 for the gravity model) and we use this for the remaining analyses. If we consider the radiation and gravity models to be nested (under the assumption that population size within a given area is uniformly distributed), we use can the likelihood ratio test to formally test the model fit (*P* < 0.05). The optimisation algorithm was not strongly influenced by the starting values, with each initial parameter set yielding very similar final estimates.

**Table 2:** Optimised Estimates for Components of the Spatial and Non-Spatial Models and the Poisson Mixed-Effects Model Coefficients.

	Non-spatial model	Distance model	Gravity model	Radiation model
**Spatial model components**				
Distance power (*γ*)	—	1.89(1.68–2.11)	1.75(1.31–2.19)	—
Source population (*μ*)	—	—	2.08(0.53–3.63)	—
Destination population (*ν*)	—	—	2.71(1.25–4.17)	—
Within LGA transmission (*α*_*1*_)	2.81(1.02–4.59)	3.01(0.68–5.34)	1.08(0.20–1.97)	3.77(0.52–7.03)
**B**etween LGA transmission (*α*_*2*_)	—	1.04(-0.65–2.74)	1.00(-0.47–2.48)	—
- Commuter flux susceptible to infected LGA	—	—	—	0.65(-0.49–1.78)
- Commuter flux infected to susceptible LGA	—	—	—	4.63(1.05–8.22)
Long-range transmission (*α*_*3*_)	0.002(0.002–0.002)	0.002(0.002–0.002)	0.001(0.001–0.001)	0.000(-0.0002–0.0002)
**Poisson mixed-effects model coefficients**				
Force of infection (*λ*)	0.52(0.47–0.56)	0.51(0.46–0.55)	0.44(0.40–0.48)	0.39(0.37–0.42)
Season**(high versus low)**	0.82(0.65–0.99)	0.82(0.65–0.99)	0.66(0.50–0.83)	0.72(0.53–0.87)
Log likelihood	-5227.799	-5225.761	-5218.819	-5022.952

The spatial model components and mixed-effects model coefficients for each model are estimated jointly using an optimisation algorithm which maximises the log likelihood returned by the mixed-effects model.

There are two key quantities estimated in the radiation model. Firstly, how much infection is exported by movement from infected LGAs to their connected networks? Secondly, how much infection is acquired by movement from uninfected LGAs to an infected LGA? The model suggests that the first quantity (exporting infection from an infected LGA) is a stronger determinant of spatial spread of the virus than the second (coefficient 4.63, 95% CI: 1.05, 8.22 compared with 0.65, 95% CI: -0.49, 1.78). The log odds of a case increase by 0.72 (95% CI: 0.53, 0.87) in the high season of poliovirus transmission relative to the low season.

**Fig 1:**
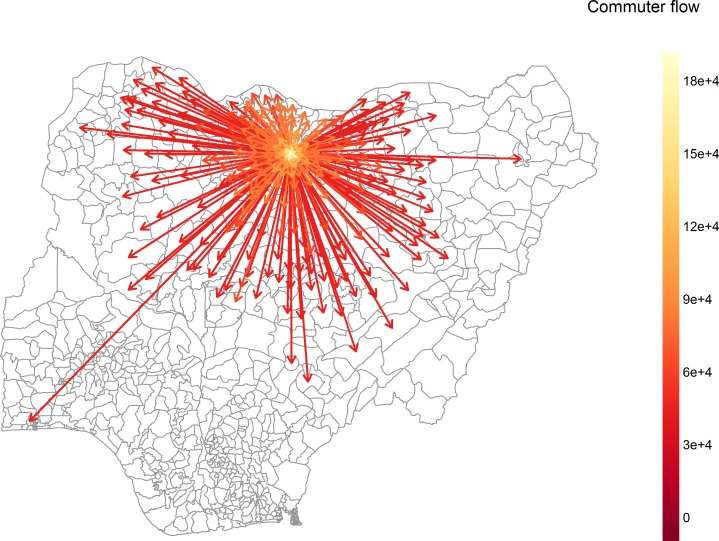
Simulated population movement under the radiation model from Kano Municipal Area, Kano state (population density 14 064.1 people per km^2^). Population movements with fewer than ten people are excluded.

**Fig 2:**
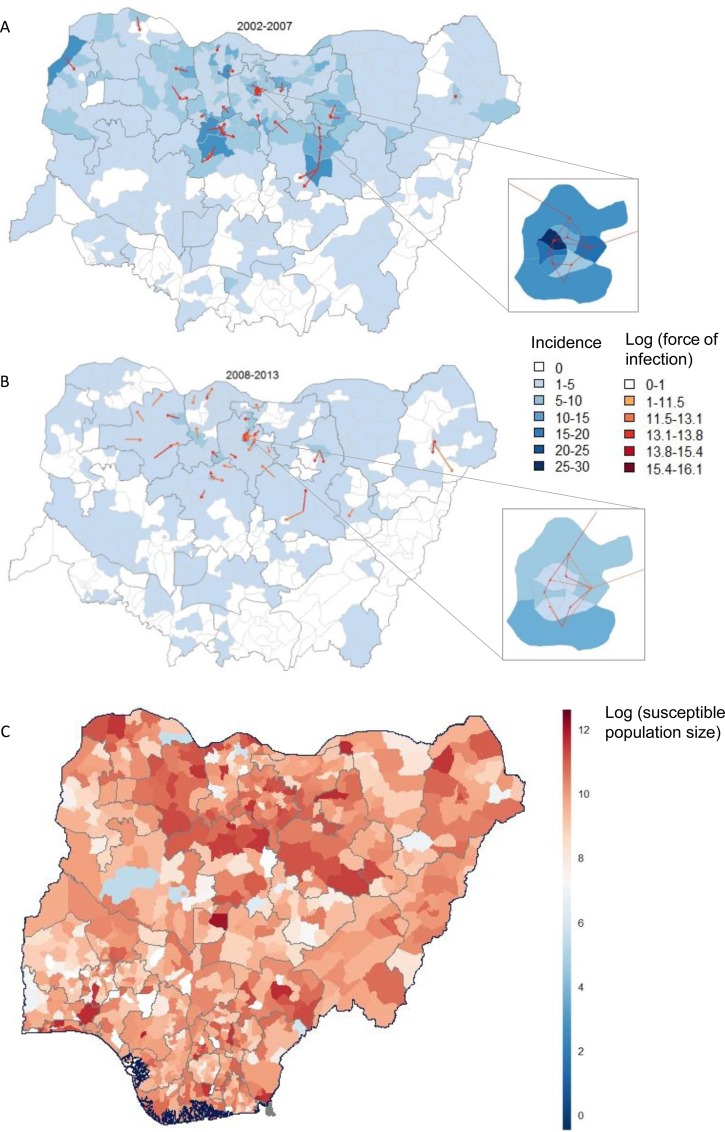
**The most probable infection pathways for the northern states of Nigeria (arrows) based on estimated population movement during 2002–2007 (A) and 2008–2013 (B). The expected number of susceptible children (aged under 15 years) during April-September 2012 based on reported vaccination records and vaccine efficacy (C).** Arrows indicating the direction of the infection pathway originate from the centrepoints of infected LGAs to the most highly connected LGAs and are colour-coded by the strength of the force of infection (A-B). The force of infection is estimated by the incidence within LGA *i* and the spatial coupling between LGAs *i* and *j*, following the radiation model. Incidence of poliomyelitis is aggregated over the time-periods (fill colours) and refers to confirmed, symptomatic cases caused by wild-type 1 poliovirus only. Inset: Kano Municipal Area and surrounding LGAs.

Population movement predicted by the radiation model for Kano Municipal Area in Kano state is illustrated in Fig 1. The predicted pathways of high transmission show highly localised spread, dominated by short-range infection paths (Fig 2, S2–S4 Figs). We present here only the transmission pathways linking LGAs in northern states of Nigeria as incidence of poliomyelitis in the southern states is relatively rare (123 cases in southern states and 3414 cases in northern states during the study period). The inset maps in Fig 2A and 2B show that transmission between Kano Municipal Area and the surrounding LGAs is highly probable and the strong inter-connectivity of these areas is likely to support continued poliovirus transmission in the absence of high population immunity.

The radiation model predicts that, given the size of the susceptible population during April to September 2013, 67.7% of secondary spread resulting from two cases in Kano Municipal Area would occur within Kano and in neighbouring Gwale and Tarauni LGAs. A further eight highly connected LGAs located on average 323.7km from Kano Municipal Area have predicted caseloads of >0.25. In this instance, a mop-up vaccination campaign targeting approximately 500,000 children in Kano and the three neighbouring LGAs could prevent any further cases emerging. However, given the high connectivity of LGAs in this area (Fig 2A and 2B insets), protecting all children within highly connected areas would be recommended.

### Validation

**Fig 3:**
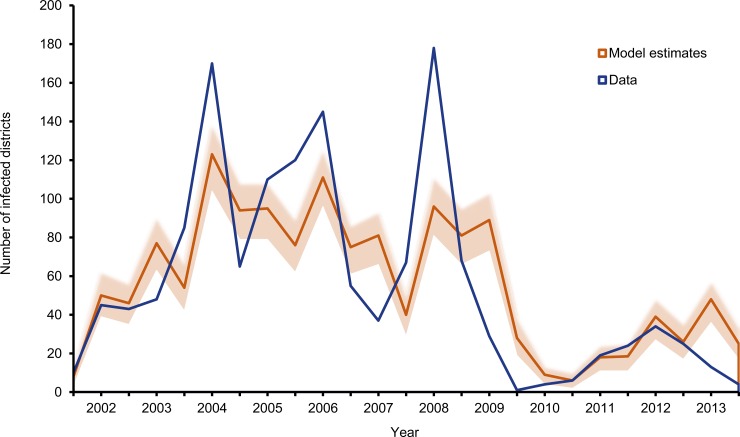
The expected number of LGAs (of 774) reporting at least one case of poliomyelitis during each six month period. The shaded area represents 95% of the distribution of outcomes using 1000 simulations of the radiation model with the actual number of LGAs reporting a case overlaid in blue.

We first simulated 1000 datasets using the best-fitting model and calculated the mean number of LGAs predicted to experience an outbreak for each simulation (Fig 3). A comparison of these simulations with the observed values indicated reasonable agreement (mean R^2^ value 0.62 [95% CI: 0.54, 0.70]). However, the model failed to capture the short-term dynamics and lagged behind the observed data, underestimating the number of infected LGAs at the start of a peak. Secondly we examined the geographic distribution of cases in 1000 simulated datasets at each time point, comparing the performance of the model in identifying which LGAs would become infected with the observed data. The geographic distributions of cases were well-estimated with an average AUC of 0.81 (SE 0.02) suggesting that it performs well in estimating the probability and location of outbreaks (S5 Fig).

## Discussion

The results from the four multivariate models all conclude that within-LGA transmission is highly predictive of future transmission, in agreement with earlier studies of poliovirus transmission in Nigeria [10–12]. All three spatial models (distance, gravity and radiation) are strongly predictive of poliovirus risk. Our study illustrates the highly localised transmission dynamics of poliovirus, giving an indication of the scale of response needed to prevent an outbreak from spreading. The current WHO guidelines for the vaccination response following detection of circulating polioviruses in large population countries are to vaccinate between two million and five million children in the affected and adjacent areas [24]. The majority of predicted high probability transmission routes occur between spatially contiguous LGAs and so a response policy protecting all neighbouring LGAs surrounding an infected area would likely be effective in preventing spread. This scale of response targeting children in all highly linked areas would be necessary even if only one affected child is identified, as each symptomatic case can be connected with potentially hundreds of asymptomatic infections

The best fitting model of population movement to the incidence of poliomyelitis was the radiation model. This parameter-free model has other advantages, including removing the need to estimate the population and distance exponents (*μ*, *ν* and *γ*), which require some prior knowledge of mobility patterns, frequently absent in areas most affected by infectious diseases. An additional advantage of the radiation model over the gravity model is the ability to distinguish migration into or away from a population centre. Here the radiation model predicts that people travelling from infected LGA *j* are more likely to infect uninfected LGA *i*, than people travelling from *i*, becoming infected in LGA *j* and then returning home. This is a particularly interesting point and worthwhile of additional investigation as Temporary Recommendations on vaccination of travellers from infected areas are issued by the International Health Regulations Emergency Committee in light of the declaration of wild poliovirus spread as a public health emergency of international concern [25].

Our approach has a number of limitations. Firstly, we only expect to see one symptomatic case for every 200 infections; therefore, some LGAs will have circulating poliovirus that is not picked up by routine surveillance. We assume here that the total number of infections (symptomatic plus asymptomatic) is proportional to the number of cases of paralysis, as the proportion of infections that cause paralysis is fairly constant. The model fits the post-2010 data much better and this may in part be due to improvements in surveillance in recent years (median R^2^ for 2002–2005 was 0.66 [95% CI 0.51–0.79] compared with 0.89 [0.46–0.99] for 2011–2012). Secondly, aggregating population immunity estimates for children under five years of age may not adequately capture short time-scale dynamics. Estimating immunity in children up to three years of age would perhaps better reproduce the temporal trends in immunity, but the small numbers of non-polio AFP cases in this age group in many LGAs made it difficult to obtain reliable immunity estimates. Thirdly, we rely on recall of vaccination history for children by their carers when we estimate population immunity. These estimates may therefore be subject to errors, which have been described elsewhere, although they have been shown to be highly predictive of an outbreak at the population-level [11, 12, 26]. Finally, we have relied on a simple model of population movement, not empirical data. Mobility data in sub-Saharan Africa is generally limited, however, using mobile phone call records in combination with census data to track individual movement patterns, previously implemented in West Africa, offers an interesting opportunity [27, 28].

Maps of wild poliovirus outbreaks in Nigeria revealing genetic clusters support our findings, showing markedly clustering of related genotypes [29]. Each of the three main reservoirs (Northwest, North Central, and Northeast Nigeria) supports distinct clusters with some transmission between adjacent areas. Integrating this work with genetic sequence data would provide a more thorough understanding of the spatial dynamics, helping to inform guidelines for mop-up campaigns following an outbreak.

Our findings emphasize the need for focussed responses to outbreaks, prioritising high coverage in LGAs along high transmission pathways. The recent successes in the polio eradication efforts in Nigeria during 2014 and 2015, driven by significant improvements in SIA campaign quality and micro planning to increase vaccine coverage in poor performing LGAS, demonstrate that eradication of polio in this region is possible. The current Nigeria Polio Eradication Emergency Plan identifies targeted micro planning as a strategic priority [23]. This form of concentrated response to outbreaks, along with continued improvements in childhood immunity against poliomyelitis could finally achieve Nigeria’s goal of a polio-free society.

## Supporting Information

S1 Fig Population density (population size / area km2) in Nigeria with northern states highlighted in red. (TIF)

S2 Fig **(*i-iii*). The most probable infection pathways for the northern states of Nigeria (arrows) based on estimated commuter flows during 2005–2007**. Arrows indicating the direction of the infection pathway originate from the centrepoints of infected LGAs to the most highly connected LGAs and are colour-coded by the strength of the force of infection. The force of infection is estimated by the incidence within LGA *i* and the spatial coupling between LGAs *i* and *j*, following the radiation model. Incidence of poliomyelitis is aggregated over the time-periods (fill colours) and refers to confirmed, symptomatic cases caused by wild-type 1 poliovirus only. Inset: Kano Municipal Area and surrounding LGAs.
(TIF)

S3 Fig **(*i-iii*). The most probable infection pathways for the northern states of Nigeria (arrows) based on estimated commuter flows during 2008–2010.** Arrows indicating the direction of the infection pathway originate from the centrepoints of infected LGAs to the most highly connected LGAs and are colour-coded by the strength of the force of infection. The force of infection is estimated by the incidence within LGA *i* and the spatial coupling between LGAs *i* and *j*, following the radiation model. Incidence of poliomyelitis is aggregated over the time-periods (fill colours) and refers to confirmed, symptomatic cases caused by wild-type 1 poliovirus only. Inset: Kano Municipal Area and surrounding LGAs.
(TIF)

S4 Fig **(*i-iii*). The most probable infection pathways for the northern states of Nigeria (arrows) based on estimated commuter flows during 2011–2013.** Arrows indicating the direction of the infection pathway originate from the centrepoints of infected LGAs to the most highly connected LGAs and are colour-coded by the strength of the force of infection. The force of infection is estimated by the incidence within LGA *i* and the spatial coupling between LGAs *i* and *j*, following the radiation model. Incidence of poliomyelitis is aggregated over the time-periods (fill colours) and refers to confirmed, symptomatic cases caused by wild-type 1 poliovirus only.
(TIF)

S5 Fig Receiver operating characteristic (ROC) curve illustrating the performance of the model in identifying outbreaks in local government areas (LGA) averaged across each six month interval between 2001 and 2013. Predictions of outbreaks were generated for each LGA during each six month time period and a ROC curve produced for each set of predictions. The variation around the average curve (green line) is depicted using 95% confidence intervals around the average. The area under the curve (AUC) and the standard error of the AUC are 0.81 and 0.02 respectively.
(TIF)

## Abbreviations

LGA: local government area
SIA: supplementary immunisation activities
OPV: oral poliovirus vaccine
AFP: acute flaccid paralysis
ROC: receiver operating characteristic
AUC: area under the curve
