# Supplementary figures and images for: Spatial Dynamics and High Risk Transmission Pathways of Poliovirus in Nigeria 2001-2013
Source: PLoS One. 2016 Sep 26;11(9):e0163065. doi: 10.1371/journal.pone.0163065 (PMC5036822; doi:10.1371/journal.pone.0163065)

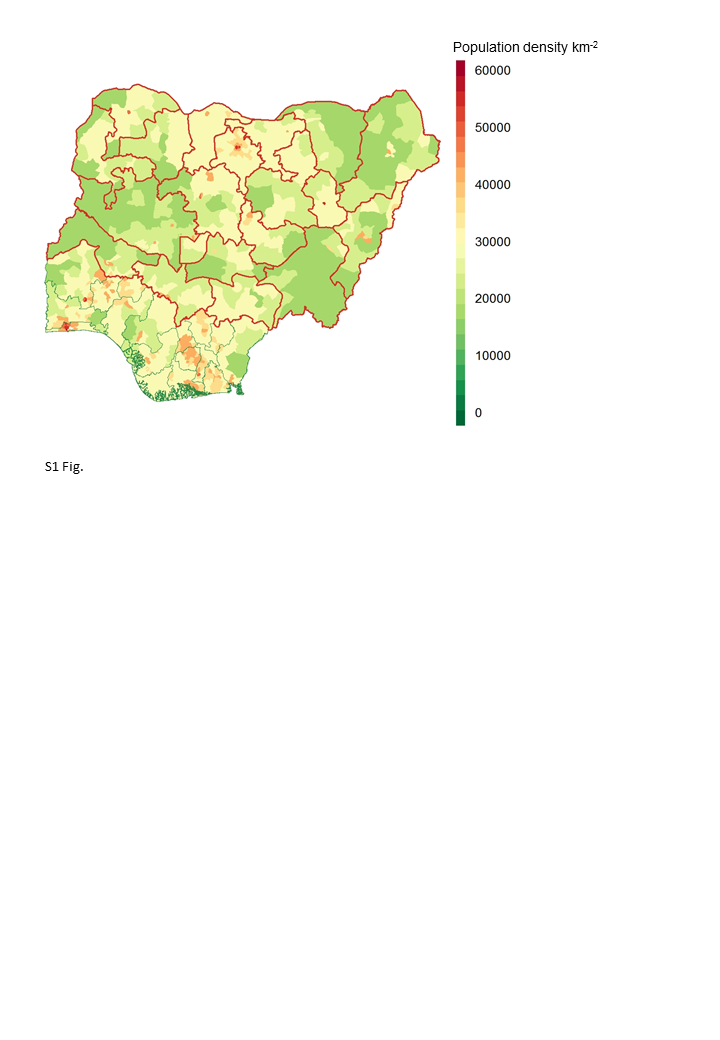

Supplement: S1 Fig — (TIF) [file pone.0163065.s001.tif]

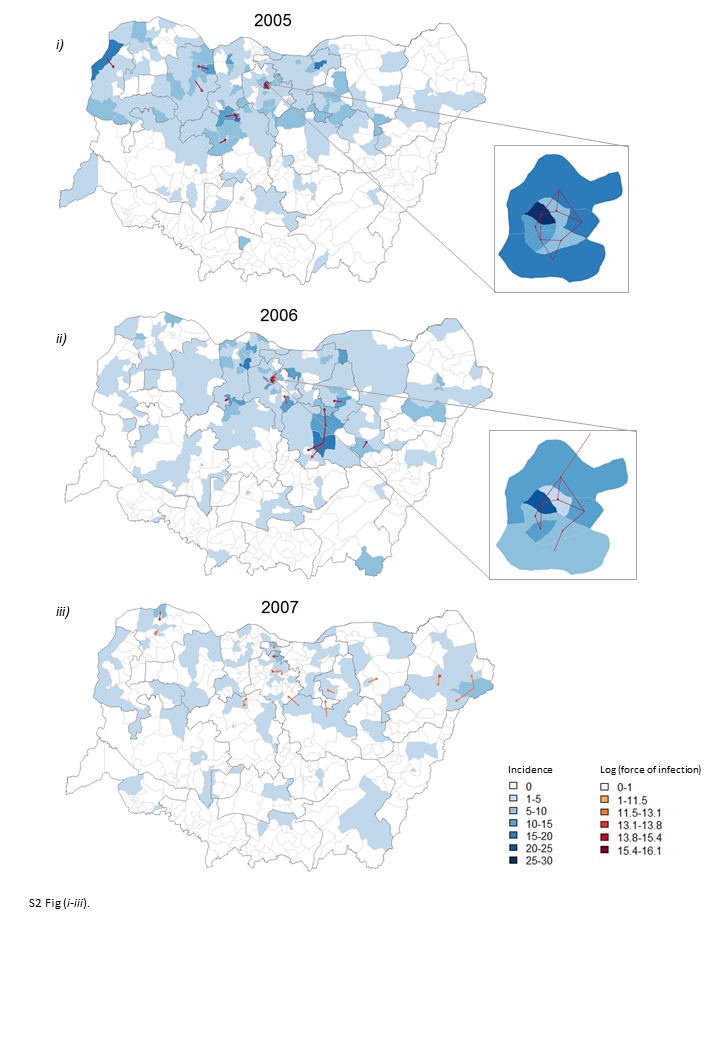

Supplement: S2 Fig — (i-iii). The most probable infection pathways for the northern states of Nigeria (arrows) based on estimated commuter flows during 2005–2007. Arrows indicating the direction of the infection pathway originate from the centrepoints of infected LGAs to the most highly connected LGAs and are colour-coded by the strength of the force of infection. The force of infection is estimated by the incidence within LGA i and the spatial coupling between LGAs i and j, following the radiation model. Incidence of poliomyelitis is aggregated over the time-periods (fill colours) and refers to confirmed, symptomatic cases caused by wild-type 1 poliovirus only. Inset: Kano Municipal Area and surrounding LGAs. (TIF) [file pone.0163065.s002.tif]

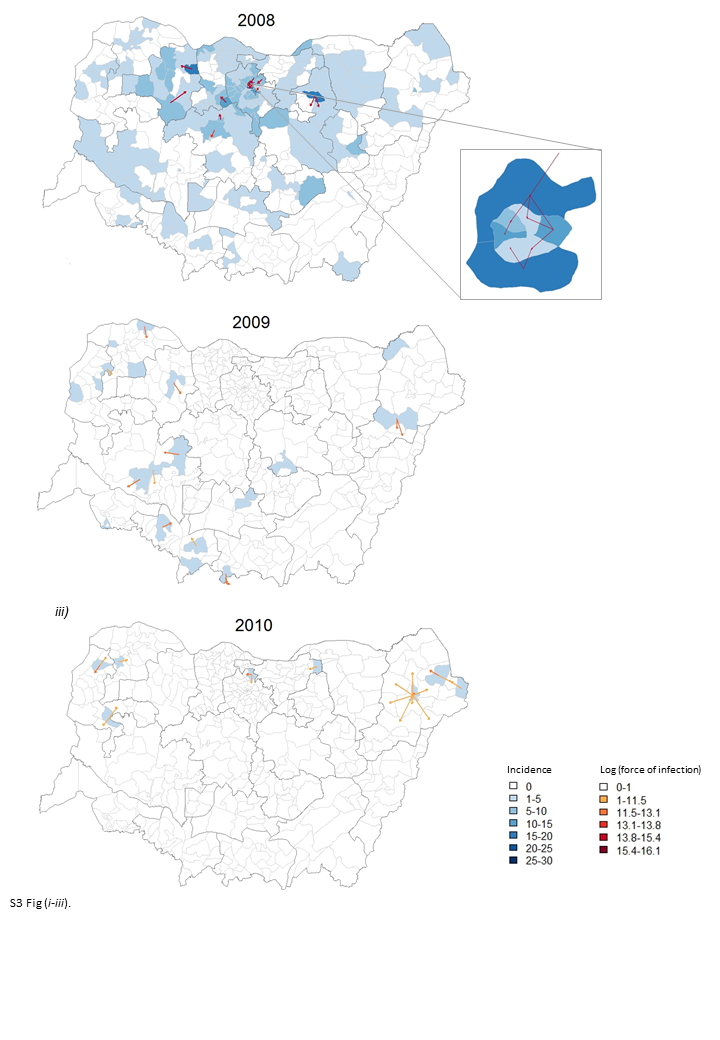

Supplement: S3 Fig — (i-iii). The most probable infection pathways for the northern states of Nigeria (arrows) based on estimated commuter flows during 2008–2010. Arrows indicating the direction of the infection pathway originate from the centrepoints of infected LGAs to the most highly connected LGAs and are colour-coded by the strength of the force of infection. The force of infection is estimated by the incidence within LGA i and the spatial coupling between LGAs i and j, following the radiation model. Incidence of poliomyelitis is aggregated over the time-periods (fill colours) and refers to confirmed, symptomatic cases caused by wild-type 1 poliovirus only. Inset: Kano Municipal Area and surrounding LGAs. (TIF) [file pone.0163065.s003.tif]

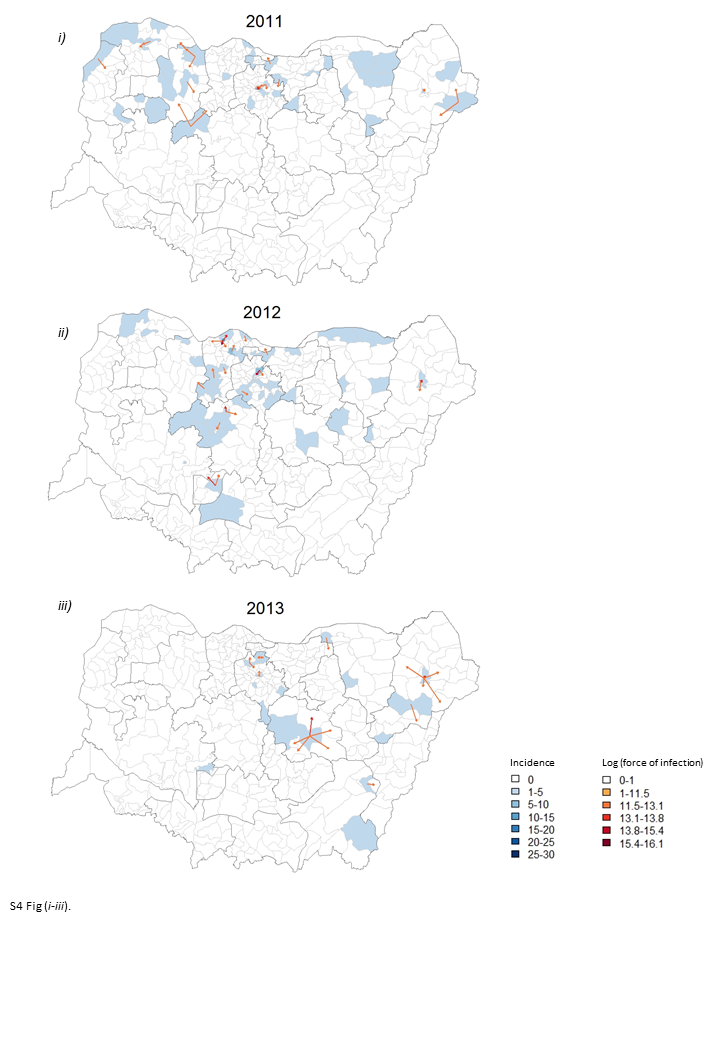

Supplement: S4 Fig — (i-iii). The most probable infection pathways for the northern states of Nigeria (arrows) based on estimated commuter flows during 2011–2013. Arrows indicating the direction of the infection pathway originate from the centrepoints of infected LGAs to the most highly connected LGAs and are colour-coded by the strength of the force of infection. The force of infection is estimated by the incidence within LGA i and the spatial coupling between LGAs i and j, following the radiation model. Incidence of poliomyelitis is aggregated over the time-periods (fill colours) and refers to confirmed, symptomatic cases caused by wild-type 1 poliovirus only. (TIF) [file pone.0163065.s004.tif]

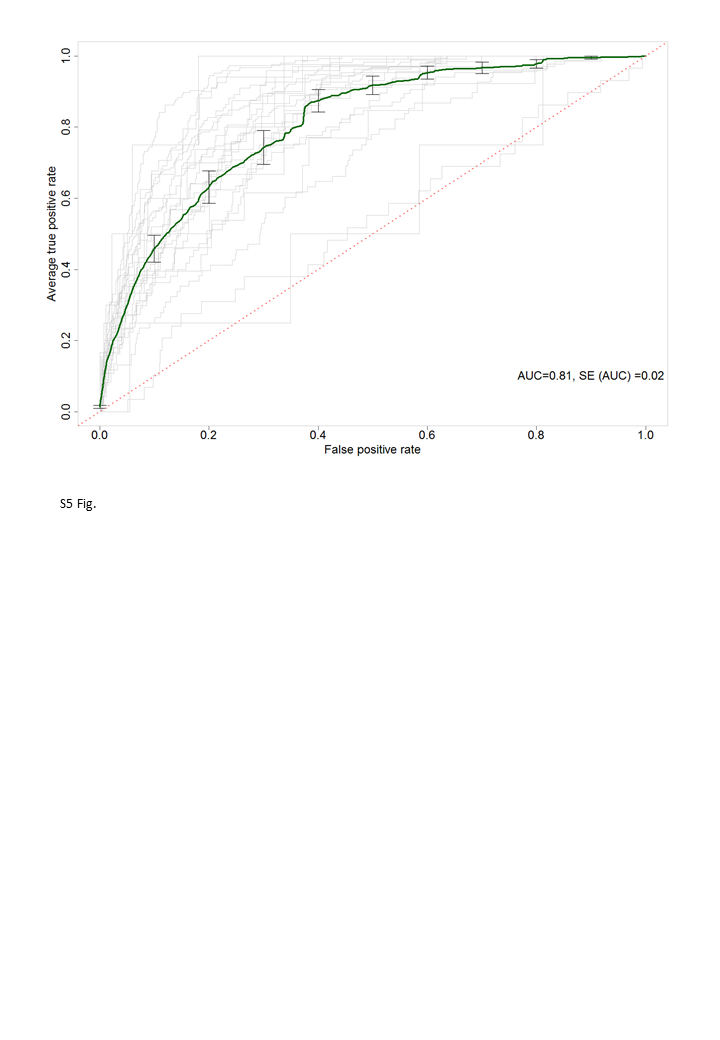

Supplement: S5 Fig — Predictions of outbreaks were generated for each LGA during each six month time period and a ROC curve produced for each set of predictions. The variation around the average curve (green line) is depicted using 95% confidence intervals around the average. The area under the curve (AUC) and the standard error of the AUC are 0.81 and 0.02 respectively. (TIF) [file pone.0163065.s005.tif]
